# Recent Developments and Future Perspectives of Molybdenum Borides and MBenes

**DOI:** 10.1002/advs.202308178

**Published:** 2024-03-25

**Authors:** Chandra Sekhar Rout, Pratik V. Shinde, Abhinandan Patra, Sang Mun Jeong

**Affiliations:** ^1^ Centre for Nano and Material Sciences Jain Global Campus Jain (Deemed‐to‐be University) Kanakapura Road Bangalore Karnataka 562112 India; ^2^ Department of Chemical Engineering Chungbuk National University Cheongju Chungbuk 28644 Republic of Korea; ^3^ Department of Molecular Sciences and Nanosystems Ca' Foscari University of Venice Via Torino 155 Mestre 30172 Italy

**Keywords:** 2D materials, catalysis, energy storage, MBene, metal boride

## Abstract

Metal borides have received a lot of attention recently as a potentially useful material for a wide range of applications. In particular, molybdenum‐based borides and MBenes are of great significance, due to their remarkable properties like good electronic conductivity, considerable stability, high surface area, and environmental harmlessness. Therefore, in this article, the progress made in molybdenum‐based borides and MBenes in recent years is reviewed. The first step in understanding these materials is to begin with an overview of their structural and electronic properties. Then synthetic technologies for the production of molybdenum borides, such as high‐temperature/pressure methods, physical vapor deposition (PVD), chemical vapor deposition (CVD), element reaction route, molten salt‐assisted, and selective etching methods are surveyed. Then, the critical performance of these materials in numerous applications like energy storage, catalysis, biosensors, biomedical devices, surface‐enhanced Raman spectroscopy (SERS), and tribology and lubrication are summarized. The review concludes with an analysis of the current progress of these materials and provides perspectives for future research. Overall, this review will offer an insightful reference for the understanding molybdenum‐based borides and their development in the future.

## Graphical abstract

Molybdenum‐based borides and MBenes have gained renowned interest due to their remarkable diversity in structure and topology, leading to unique physical and chemical properties such as tunable electrical conductivity, considerable stability, high surface area, and environmental harmlessness. This review aims to provide a comprehensive overview of the recent development and future perspectives of molybdenum borides and MBenes for various applications.

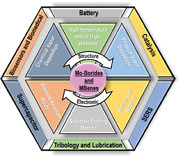

## 1 Introduction

Recently, transition metal borides (TMBs) have gained renowned interest due to their remarkable diversity in structure and topology leading to unique tunable physical and chemical properties.^[^ ^1^, ^2^, ^3^ ^]^ Most of the TMBs share M─B bonds with the strong covalent component and these bonds are stronger than those in the case of transition metal nitrides and carbides.^[^ ^4^ ^]^ Further, due to the existence of strong and highly covalent B─B bonds, TMBs exhibit numerous compositions ranging from M_3_B to MB_66_ with varying characteristics from metallic to semiconducting states. Due to their diverse compositions and crystal structures, TMBs have a wide range of interesting electronic ground states, thus featuring high chemical, mechanical, magnetic, and catalytic characteristics, thermal durability, thermoelectricity, structural complexity, and superconductivity.^[^ ^5^, ^6^ ^]^ Therefore, as compared to other non‐metal atoms (e.g., S, O, N) in the corresponding compounds of sulfides, oxides, and nitrides, boron atoms in metal borides have several superior properties due to the chemical nature of boron.^[^ ^3^ ^]^ Boron is a metalloid element with an intermediate electronegativity (2.04 in the Pauling scale) between metals and non‐metals. Due to this reason, a large variety of bond types in borides such as metallic bonds M─M/M─B, ionic bond M─B, and covalent bonds B─B are possible.^[^ ^7^ ^]^ Boron becomes electron deficient because there is less number of valence electrons available than valence orbitals, allowing it for a wide variety of linking patterns in boride lattices.^[^ ^8^ ^]^ In addition, compared to the phosphorization and sulfurization processes, boride synthesis produces significantly lower harmful gases and is, therefore, more environmentally friendly. Therefore, TMBs offer excellent potential for growing areas of research and many discoveries due to unique boron chemistry and the possibility of making the diversity of boride composition and structure. 2D transition metal borides, abbreviated MBenes, are produced by selectively etching the A layer from multi‐layered structure (MAB phases).^[^ ^9^, ^10^, ^11^ ^]^ The M─A bond is metallic, whereas the M─B bond exhibits mixed covalent/metal/ionic characteristics. The difference between MBenes and MXenes appears to be the substitution of boron in the carbon and/or nitrogen sites of MXenes. However, because of variations in the stoichiometry and structural transitions, the MAB‐MBenes cannot be directly linked to the MAX‐MXenes combination. MBenes show high elastic modulus and good mechanical stability.^[^ ^10^, ^12^, ^13^, ^14^, ^15^ ^]^

Owing to the Pt‐like d‐band electronic structure and multiple oxidation states, Mo‐based materials display high electrical conductivity, optimal adsorption of intermediate, flexibility, strength, optoelectronic properties, etc. making them ideal candidates for various applications in sensing, catalysis, energy harvesting, electronics, energy storage, ion transport, superconductivity and hydrogen storage etc.^[^ ^1^, ^11^, ^16^ ^]^ In the past few years, 2D metal borides with covalent networks have also enticed great interest since most of them are metallic leading to be highly conductive for fast electron transport and abundant catalytically active edges guarantee higher electrocatalytic activity than the corresponding bulk. As a d‐block element with multiple oxidation states, molybdenum‐based borides show properties such as low cost, facile synthesis, structural diversity, high chemical stability, corrosion resistance, high hardness, and capability to replace precious metal catalysts.^[^ ^17^, ^18^, ^19^, ^20^ ^]^ Molybdenum‐based borides (molybdenum borides) are found in different stoichiometries and crystal structures namely α‐MoB, Mo_2_B, β‐MoB, α‐MoB_2_, β‐MoB_2_, Mo_2_B_4_, and Mo_2_B_5_, etc.^[^ ^5^, ^19^, ^20^, ^21^, ^22^ ^]^ The abundant structures of Mo borides represent interesting chemical and mechanical properties which are established by the rearrangement and proportion of boron and molybdenum atoms in a unit cell.^[^ ^23^, ^24^ ^]^ Due to the peculiar crystal structure and presence of unsaturated edge sites, molybdenum boride‐based nanomaterials have both good conductivity and abundant catalytically active sites rising from the serious electron deficiency of boron atoms.

**Figure 1:**
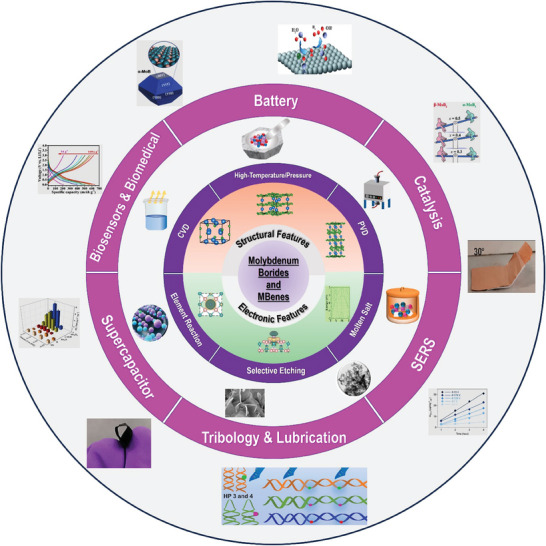
Schematic representation of preparation, properties, and recent developments of molybdenum‐based borides and MBenes.

In this timely review, we aim to provide a comprehensive overview of the recent development and future perspectives of molybdenum borides and MBenes for various applications (**Figure** **1**). This review will undoubtedly aid researchers in understanding the general state of these materials and will stimulate more effort devoted to this field. We begin with a brief introduction of molybdenum borides borides and MBenes including their structures, electronic and physicochemical properties, and synthesis approaches. Then, we present the most recent advancements in a variety of applications for these novel materials, such as energy storage (battery and supercapacitors), energy conversion, catalysis, lubrication, biosensor and surface‐enhanced Raman spectroscopy (SERS), etc. In the end, comments and viewpoints on current difficulties, challenges, and future perspectives of materials are presented.

## 2 Typical Structure and Electronic Features

### 2.1 Structural Features

**Figure 2:**
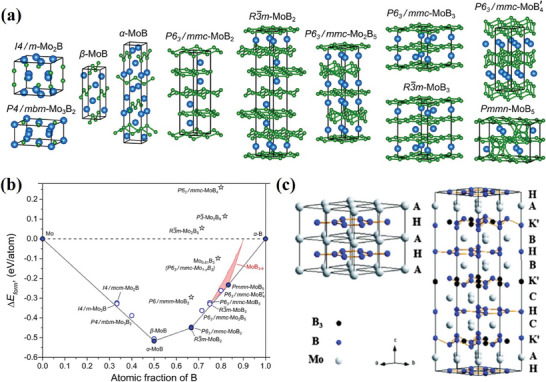
a) The Mo‐B phases' crystal structures obtained by an evolutionary crystal structure search; b) Calculated formation energies for the Mo‐B system. Stable and metastable phases are indicated by filled and hollow circles, respectively. The formation energy of the Mo‐B structures suggested in the experimental investigations is displayed by stars. The shaded area portrays the energies of the formation of boron‐rich MoB_x_ phases with 3 ≤ x ≤ 9, obtained using a parameterized lattice model. Reprinted with permission.^[^ ^26^ ^]^ Copyright 2020. American Chemical Society. c) Crystal structures of α‐MoB_2_ (*P6/mmm*) structure with …AHAHAH… stacking sequence and β‐MoB_2_ (*R‐3m*) structure with …AHA K′ BHB K′ CHC K′ A… stacking sequence. Reprinted with permission.^[^ ^30^ ^]^ Copyright 2013. Royal Society Chemistry.

Molybdenum borides can be classified into three major types depending on the Mo and B molar ratios:^[^ ^25^ ^]^ i) When the molar ratio Mo/B <0.5, the boron‐rich borides show the structure of a 3D framework without metal‐metal bonds; ii) For the molar ratio Mo/B >2, the metal‐rich borides exhibit metal structure without boron‐boron bonds; iii) For the moderate molar ratio (0.5 ≤ M/B ≤ 2), many bonds are possible and usually boron atoms construct a low dimensional substructure (boron dumbbells, boron chains, and boron layers). **Figure** **2**a shows the crystal structures of the Mo‐B system with the information about their space groups and Figure 2b summarizes the calculated energies of formation of the stable and metastable phases.^[^ ^26^, ^27^, ^28^ ^]^ For the metal‐rich molybdenum boride, the Mo atoms are bonded together to form a 3D metal skeleton, whereas the boron atoms/pairs are dispersed in the metal skeleton by Mo─B bonds.^[^ ^1^, ^3^ ^]^ In mono borides such as Mo─B, the metal atoms are arranged similarly to the elemental metals, while the boron atoms are inserted into the gap causing some expansion of the lattice. MoB and Mo_2_B_2_ are known to belong to the MBene family and are reported to be prepared from MoAlB by selective etching of Al layers.^[^ ^29^ ^]^ MBenes are the class of orthorhombic and hexagonal 2D transition metal borides obtained from MAB phases with a chemical formula of M_n_B_n‐2_. In the case of Mo‐based MBenes, the crystal structure is the alternate stacking of the metal (Mo) and B layers and they can be classified into two categories: orthorhombic MBenes (ortho‐MBenes) and hexagonal MBenes (hex‐MBenes). Molybdenum diborides exist in two forms known as α‐MoB_2_ and β‐MoB_2_. In α‐MoB_2_, boron atoms are arranged into 2D graphitic boron layers whereas in β‐MoB_2_ they form puckered boron layers (Figure 2c).^[^ ^2^, ^3^, ^30^ ^]^ α‐MoB_2_ is an AlB_2_‐type crystal phase structure, with alternating planar sheets of metals and boron atoms in sequences AHAHAHAH… β‐MoB_2_ is considered as the Mo_2_B_5_‐type structure with AHA K BHB K CHC K A… arrangements (Figure 2c), where A, B, C are close‐packed Mo layers; layers B and C are shifted by (a/3, 2a/3) and (2a/3, a/3) relative to A respectively and H is the graphite type layer constituted by B_1_ atoms. The K‐type layer is a puckered and densely packed boron network consisting of B_2_ and B_3_ atoms. In the case of boron‐rich systems [MoB_3_, MoB_4_, and MoB_5_, etc.], boron cages/clusters act as the structural units and are formed by the covalent bonds between boron atoms and connected further to form 3D boron skeleton. The metal atoms are inserted in the void of boron skeletons, which give additional valence electrons for the boron structural unit lacking electrons.

### 2.2 Electronic Features

**Figure 3:**
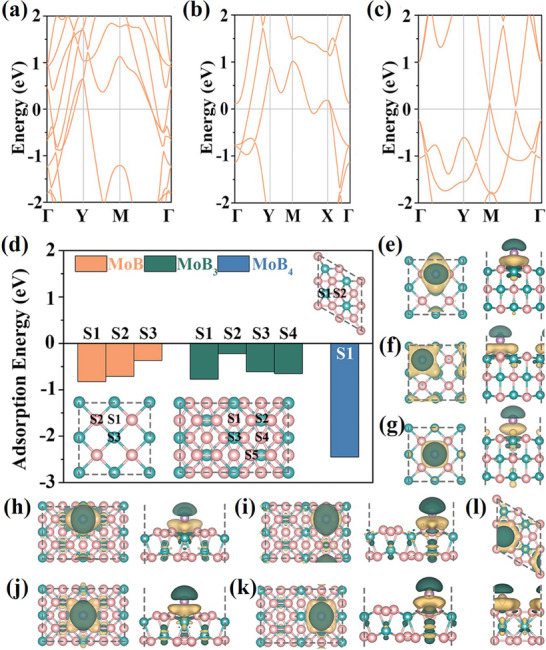
a−c) Electronic band structures of the MoB_x_ (x = 1, 3, and 4) monolayers; d) Adsorption energies of Li on the MoB_x_ (x = 1, 3, and 4) monolayers. The insets show the considered adsorption sites. Top and side views of the charge redistributions induced by the interaction of Li at the e) S1, f) S2, and g) S3 sites with the MoB monolayer, by the interaction of Li at the h) S1, i) S2, j) S3, and k) S4 sites with the MoB_3_ monolayer, and by the interaction of Li at the l) S1 site with the MoB_4_ monolayer. Green and yellow isosurfaces (isovalue: 0.01 electrons Å^−3^) represent charge depletion and accumulation, respectively. Reproduced under terms of the CC‐BY 4.0 license.^[^ ^27^ ^]^ Copyright 2022. Springer Nature.

Due to the diversity in the compositions of molybdenum borides, they can be arranged in varieties of crystal structures and possess different physical, and electronic properties. Further, their properties can be varied by non‐stoichiometry or by incorporation of impurities.^[^ ^4^, ^31^ ^]^ The calculated electronic band structures of different molybdenum boride mono layers show Fermi level crossing of several bonds indicating excellent metallic behavior of the MoB_x_ (x = 1, 3, 4 monolayers) (**Figure** **3**a−c).^[^ ^27^ ^]^ The theoretical studies have confirmed that stable adsorption of Li atoms as the impurity is possible from different positions of the MoB_x_ layers (Figure 3d). The Li adsorption resulted in charge transfers of 0.85−0.88 e^−^ demonstrating its suitability for energy storage applications. Electronic structure calculations of Mo_2_B revealed its typical metallic and non‐magnetic nature with the negligible effect of native defects, biaxial tensions, and compressions on its properties.^[^ ^32^ ^]^ Due to the low‐frequency vibrations of the Mo atoms and the electronic occupations of the Mo‐4d orbitals of tetr‐ and tri‐Mo_2_B_2_ near the Fermi level, it shows intrinsic phonon superconductivity with a superconducting transition temperature (*T*_c_) of 3.9 and 0.2 K respectively.^[^ ^33^ ^]^

## 3 Synthesis of Molybdenum Borides

Molybdenum borides can be synthesized by various physical and chemical methods including traditional solid‐state, high‐temperature, and/or high‐pressure methods. These methods are physical vapor deposition (PVD), chemical vapor deposition (CVD), element reaction route, molten salt‐assisted, and selective etching method.

### 3.1 High‐Temperature and/or High‐Pressure Methods

**Figure 4:**
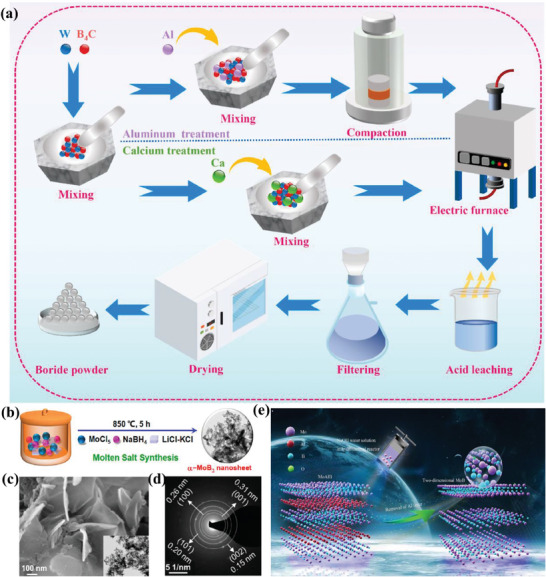
a) Schematic represents the preparation process of borides. Reprinted with permission.^[^ ^34^ ^]^ Copyright 2023. Elsevier, b) Schematic representation of the preparation of α‐MoB_2_ nanosheets by the molten salt method, c) SEM and transmission electron microscopy (TEM) images of α‐MoB_2_ nanosheets, d) Selected area electron diffraction (SAED) pattern for the α‐MoB_2_ nanosheets. Reprinted with permission.^[^ ^18^ ^]^ Copyright 2022. American Chemical Society. e) The schematic of the preparation of 2D MoB Mbene. Reprinted with permission.^[^ ^48^ ^]^ Copyright 2022. Elsevier.

Molybdenum borides (Mo_2_B, MoB, Mo_2_B_5_, MoB_4_, MoB_2_, etc.) can be synthesized by high temperature (>1200 K) and by high pressure (≈5.2 GPa) sintering (**Figure** **4**a).^[^ ^4^, ^17^, ^34^ ^]^ It is reported that by annealing the varied mixture of B/Mo precursor (molar ratio: 0.5, 1.0. 2.5, 4, etc.) in the presence of appropriate carbonization/decarbonization agent (Ca, Al, and Mg), different phases of molybdenum borides (Mo_2_B, MoB, Mo_2_B_5_, MoB_4_, MoB_2_, etc.) with controlled size can be prepared.^[^ ^35^, ^36^, ^37^, ^38^ ^]^ In these high temperature‐based approaches, boro/carbothermal reduction processes occur for the synthesis of the high entropy boride powders. However, these methods have several demerits since obtained products usually contain mixed phases with uncontrolled crystallization and large particle size.^[^ ^38^ ^]^ The element reaction route is the common method for molybdenum boride synthesis in which the sintering of molybdenum and boron mixture in vacuum or inert gas is carried out under rigorous circumstances in a high‐temperature instrument or arc‐melting chamber.^[^ ^20^, ^39^ ^]^ Chen et al. reported the synthesis of several Mo‐B phases with different crystal structures such as Mo_2_B, MoB, α‐MoB_2_, and β‐MoB_2_ at high pressure of 5.2 GPa and high temperature of 1600−1800 °C.^[^ ^20^ ^]^ Park's group employed an arc‐melting method to synthesize Mo_2_B, α‐MoB, β‐MoB, and α‐MoB_2_.^[^ ^19^ ^]^ Further, it is demonstrated that the use of Sn flux promoted the reactivity and diffusion of solid reactants leading to the formation of single‐phase Mo_2_B_4_ material.^[^ ^21^ ^]^ Nguyen et al. prepared the molybdenum boride powder by a cost effective and simple pulsed discharge of compacted crystalline and amorphous micron‐sized B powder.^[^ ^39^ ^]^ In this method, the raw material (Mo rod and B micron‐sized powder) was heated by a large pulsed current of electrical energy stored in capacitors. The vapor diffused with the vaporized/melted precursors formed the nanoparticles through a series of processes such as supersaturation, nucleation, and condensation (Figure 4b).^[^ ^39^ ^]^

### 3.2 PVD and CVD

PVD is the direct method for the synthesis of molybdenum borides without using any chemical etching and no involvement of chemical reactions.^[^ ^40^, ^41^, ^42^ ^]^ PVD method offers distinct advantages in controlling the synthesis and structure of MBenes, thereby influencing their electrical behavior. PVD‐grown MBenes typically exhibit high electron mobility and excellent conductivity due to the well‐defined crystalline structures achieved during the deposition process. Sahu et al. reported the growth of 2D MoB MBene domains in a MoAlB thin film by Al deintercalation from MoAlB in the vicinity of AlO_x_ region via direct current magnetron sputtering (DCMS).^[^ ^42^, ^43^ ^]^ CVD is one of the controlled synthesis approaches to prepare molybdenum borides by heating the borane or a mixture of boron and its oxide as the boron source, Mo metal as the metal source, and hydrogen as the carrier gas and reducing gas.^[^ ^44^, ^45^, ^46^, ^47^, ^48^ ^]^ Conversely, CVD‐grown MBenes often demonstrate superior tunability and scalability, attributed to the precise control over growth parameters and defect engineering facilitated by the chemical nature of the deposition process. Comprehending the intricate interaction between deposition methods and the consequent electrical characteristics is vital for enhancing the performance of MBene‐based electronic devices across a wide array of applications, spanning from flexible electronics to quantum computing. By this approach, Wang et al. obtained large area, ultra‐thin, and highly conducting metallic hexagonal Mo_3_B film.^[^ ^5^ ^]^ Si et al. demonstrated a chemical potential modulated strategy to realize the precise synthesis of various ultra‐high purity ultra‐thin molybdenum borides by the CVD method.^[^ ^49^ ^]^

### 3.3 Molten Salt Method

The molten salt‐assisted route is a promising approach with control over the phase formation and morphology to avoid excessive grain growth and high crystallinity.^[^ ^50^, ^51^ ^]^ Jothi et al. reported the synthesis of molybdenum mono borides (α‐MoB) and molybdenum diborides (MoB_2_ and Mo_2_B_4_) by using anhydrous Mo‐Cl_5_ and elemental boron in the presence of Sn metal precursor.^[^ ^38^ ^]^ at high temperature (800−900 °C for 8 h). Liu et al. reported the synthesis of single‐phase polycrystalline α‐MoB_2_ with nanosheet morphology through the MoCl_5_ and NaBH_4_ reaction in the LiCl‐KCl molten salt (Figure 4c).^[^ ^18^ ^]^ By changing the “B” precursor to “B” powder β‐MoB_2_ was achieved under similar experimental conditions.^[^ ^52^ ^]^

### 3.4 Selective Etching Method

**Figure 5:**
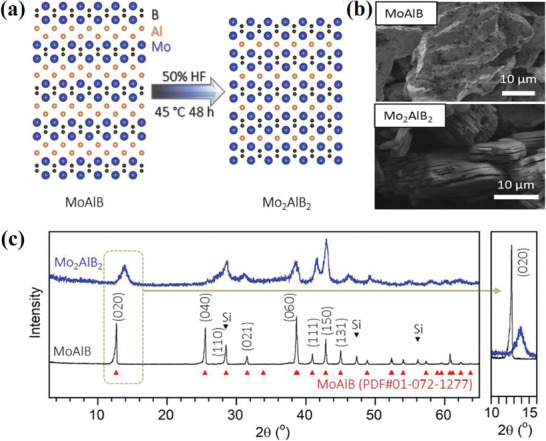
a) Schematic for the structural evolution of Mo_2_AlB_2_ from MoAlB, b) SEM of MoAlB before and after etching, c) XRD of MoAlB before and after HF treatment. Reprinted with permission.^[^ ^57^ ^]^ Copyright 2023. Wiley.

Similar to MXene synthesis from the MAX phase by etching of “A” element, MoAlB etching is reported by different etchants such as NaOH, HF, and LiF in HCl.^[^ ^46^, ^53^, ^54^ ^]^ However, it is observed that the metastable phase Mo_2_AlB_2_ emerges during topochemical de‐intercalation of Al from MoAlB, and complete etching of Al is found to be difficult.^[^ ^54^ ^]^ A schematic in Figure 4d shows the fabrication process of 2D MoB MBene through a hydrothermal‐assisted alkane solution etching strategy.^[^ ^46^ ^]^ The pristine MoAlB exhibits a lamellar‐stacking structure, whereas MoB particles exhibit accordion‐like layer structure like MXene materials. It is reported that MBenes could not be directly obtained from MoAlB because of a structural difference in the Al layers between MAX and MAB phases, i.e., MAX phases contain a single Al layer while MAB has a zigzag double Al layer. Due to this reason, partially “Al” etched MBenes are represented as MoAl_1‐x_B.^[^ ^55^ ^]^ Zhou et al prepared boridene (2D molybdenum boride sheets) with ordered metal vacancies, Mo_4/3_B_2‐x_T_z_ by selective etching of aluminum and yttrium or scandium atoms from 3D in‐plane (Mo_2/3_Y_1/3_)_2_AlB_2_ and (Mo_2/3_Sc_1/3_)_2_AlB_2_ in aqueous hydrofluoric acid.^[^ ^56^ ^]^ The sheets may have a minor B deficiency, with x up to ≈0.5, in comparison to the parent phase. The surface terminations T_z_ were found to be a mixture of O, OH, and F, with z ranging from 2 to 3. Majed et al. prepared the Mo_2_AlB_2_ phase by partial selective etching of Al from the MoAlB phase using HF acid at 45 °C for 48 h (**Figure** **5**a).^[^ ^57^ ^]^ The (020) reflection shift due to etching from 12.6° (2θ) to 13.8° (2θ), signifying a reduction in d‐spacing from ≈7.0 to 6.4 Å and removal of an Al layer (Figure 5c).

## 4 Applications of Molybdenum Borides and MBenes

**Figure 6:**
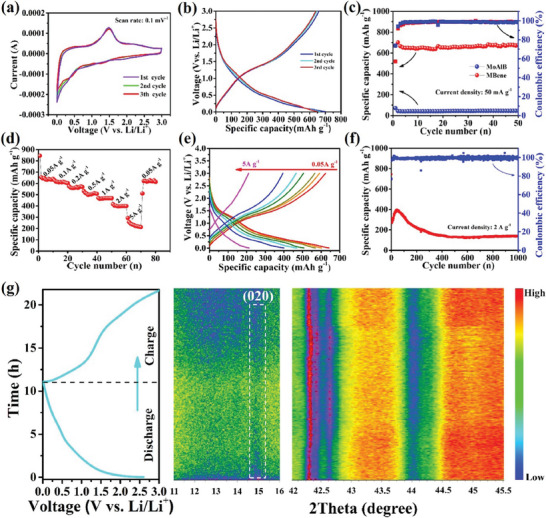
Performance of the 2D MoB MBene anodes in lithium‐ion batteries, a) Cyclic voltammetry (CV) curves at a scan rate of 0.1 mV s^−1,^ b) Charge−discharge curves at 50 mA g^−1^,c) the cycle performance of MoAlB and 2D MoB MBene at 50 mA g^−1^ d) rate capability, e) the corresponding voltage curves of 2D MoB MBene, f) long cycle performance at 2 A g^−1^,g) In situ XRD plots with the corresponding discharge−charge curve. Reprinted with permission.^[^ ^46^ ^]^ Copyright 2022. Elsevier.

Due to their several advantages including high chemical, mechanical, and thermal stability, molybdenum borides‐materials have emerged as the ideal candidate for a wide range of applications in electrochemical energy storage, catalysis, biosensors, tribology, and high‐temperature structures in the aerospace industry.^[^ ^46^, ^55^, ^57^ ^]^ In this section, we will discuss the recent applications of these materials in different fields (**Figure** **6**).

### 4.1 Energy Storage Devices

Mo borides and 2D MBenes have gained interest in energy storage applications due to their several unusual physicochemical properties with key advantages such as large surface area, active edge sites, high mechanical properties, flexibility, etc.^[^ ^20^, ^44^, ^58^, ^59^, ^60^, ^61^, ^62^ ^]^

#### 4.1.1 Battery

Theoretical predictions showed that molybdenum borides and MBenes have inherent mechanical anisotropy and metallic properties, low diffusion potential, and low open circuit voltage.^[^ ^63^ ^]^ The predicted properties of these materials suggested that they can be attractive anode materials for Li/Na ion batteries.^[^ ^63^ ^]^ Wang et al. showed that Li and Na possess negative adsorption energies when interacting with Mo_2_B, which could prevent the formation of metallic Li and Na and improve safety and reversibility when used as anodes in ion batteries.^[^ ^32^ ^]^ The diffusion barriers for Li (Na) ions in 2D Mo_2_B are reported to be 0.073 (0.069) eV and the open circuit voltages are 0.62−1.15 (0.42−1.41) V respectively. T‐type Mo_2_B is found to show high theoretical volumetric capacity (≈2424 mAhcm^−3^) with low energy barriers (0.0372 eV) as compared to the H‐type Mo_2_B.^[^ ^64^ ^]^ Similarly, MoB and MoB_3_ monolayers are found to be good anode materials of Li‐ion batteries with Li specific capacities of 670 and 418 mAhg^−1^ with low Li diffusion barriers of 0.10 and 0.13 eV respectively.^[^ ^27^ ^]^ Strong mechanical and thermal properties and high in‐plane stiffness of pristine and lithiated MoB_2_ demonstrated that it can withstand massive volume expansion at 500 K during lithiation/de‐lithiation reactions, which is remarkably beneficial for the manufacturing of flexible anodes.^[^ ^65^ ^]^ Tetragonal and trigonal Mo_2_B_2_ displayed excellent electronic conductivity and great stability for the Li and Na ion battery applications with specific capacity values in the range of ≈251 and 188 mAhg^−1^ respectively.^[^ ^66^, ^67^ ^]^ Recently, experimental results on molybdenum boride and MBenes‐based materials are demonstrated as promising candidates for the anodes of Li‐ion batteries.^[^ ^68^, ^69^, ^70^ ^]^ 2D MoB MBene etched from MoAlB exhibited a reversible specific capacity of 144.2 mAhg^−1^ after 1000 cycles at the current density of 2 Ag^−1^.^[^ ^46^ ^]^ Figure 6 shows the electrochemical performance of MBene‐based anodes in Li‐ion batteries. Ex situ X‐ray diffraction (XRD) and field emission scanning electron microscopy (FESEM) analysis of the electrodes after long cycling studies showed no noticeable change in the material in terms of its crystal structure and morphology. In situ, XRD results during the charging/discharging processes confirmed that MBenes are not electrochemically active and the formation of Li‐MoB phase occurs during lithiation/de‐lithiation (Figure 6). The Mo_2_AlB_2_ shows a higher specific capacity (593 mAhg^−1^ at 200 mAg^−1^ after 500 cycles than MoAlB electrodes.^[^ ^57^ ^]^ It is further confirmed that surface redox reactions are responsible for Li storage in MoAlB_2_ rather than intercalation or conversion processes. Due to the good hydrophilic properties and good wettability toward electrolytes of MoB, the Li‐S batteries based on these electrodes displayed impressive electrochemical performance with a high capacity of 1253 mAhg^−1^ and a long life span (1000 cycles).^[^ ^68^ ^]^ **Table** **1** summarizes the battery applications of different Mo borides and MBenes.^[^ ^46^, ^57^, ^68^, ^69^ ^]^

**Table 1:** Recent literature on the battery and supercapacitor applications of molybdenum borides and MBenes.

Application/Materials	Performance			Ref.
Battery	Ion battery type	Reversible capacity (mAhg^−1^)	Rate and cycling capability
MoB	Li	701 mAhg^−1^ at 50 mAg^−1^	144.2 mAhg^−1^ at 2 Ag^−1^ after 1000 cycles	[46]
MoAl_1‐x_B	Li	460 mAhg^−1^ at 20 mAg^−1^	209 mAhg^−1^ at 1 Ag^−1^ after 500 cycles	[56]
MoB	Li‐S	1253 mAhg^−1^ at 0.5 C	1192 mAhg^−1^ at 0.2 C after 350 cycles	[68]
Mo‐MoB	Li‐S	1188 mAhg^−1^ at 0.2 C	672 mAhg^−1^ at 0.2 C after 50 cycles	[69]

Supercapacitor	Type of supercapacitor device	Capacitance	Energy density at power density	Cycle test retention (%)/cycles	
MoB	AC filtering capacitors	702 µFcm^−2^, Negative phase angle ≈ 54.8° at 120 Hz	–	–	[70]
MoB	All‐solid‐state supercapacitor	741.6 mFcm^‐2^ at 1 mVs^−1^; stable performance even at 90° bending	24.65 µWhcm^−2^ @ 2mWcm^−2^	80.2%/ 5000	[71]
MoB	Asymmetric supercapacitor	445 Fg‐1 at 1Ag^−1^ with 1.4 working window	14 Whkg^−1^ @ 16.8 kWkg^−1^	98%/ 2000	[72]

#### 4.1.2 Supercapacitors

**Figure 7:**
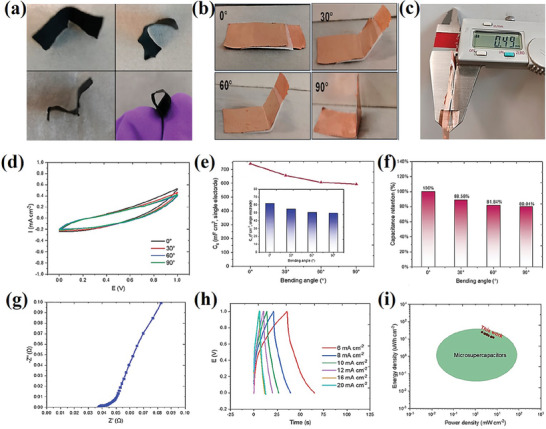
Fabrication and electrochemical measurements for 1/24‐MoAl_1−x_B all‐solid‐state supercapacitor (ASSS), a) Photographs of a 1 × 1.5 cm^2^ film electrode bent to various shapes, b) Photographs of ASSS bent to different bending angles (0, 30, 45, and 90°), c) Thickness of the fabricated ASSS, d) CV curves under different bending angles, e) capacitance under different bending angles, f) capacitance retention of the single electrode, g) Nyquist plots, h) Galvanostatic charge‐discharge (GCD) curves, i) Ragone plots. Reprinted with permission.^[^ ^71^ ^]^ Copyright 2023. American Chemical Society.

To date, only a limited number of MBenes have been employed as electrode materials for supercapacitors. However, Mo_2_B_2_ and its derivatives show promise for energy storage applications due to their notable properties such as high conductivity, electrochemical surface area, and surface roughness. This suggests that Mo_2_B_2_‐based materials could be particularly effective in enhancing the performance of supercapacitors by facilitating efficient charge transfer and storage processes. Partially “Al” etched MoAlB nanosheets are reported to show good energy storage performance when used as the electrodes of supercapacitors (Table 1).^[^ ^70^, ^71^, ^72^ ^]^ 2D MBenes applied as the pseudocapacitive filter electrochemical supercapacitor electrode material exhibited excellent AC filtering performance with a specific capacitance of 702 mFcm^−2^ under the AC condition of 120 Hz and a negative phase angle of 54.8°.^[^ ^70^ ^]^ All‐solid‐state supercapacitors based on multilayered MBenes delivered significant capacitance, high energy density, and power density even at a 90° bending angle (**Figure** **7**).^[^ ^71^ ^]^ Vinoth et al. demonstrated the asymmetric supercapacitors based on MoB nanosheets and activated carbon as the electrodes with 1 M H_2_SO_4_ and 1 M Na_2_SO_4_ as the electrolytes.^[^ ^72^ ^]^ The asymmetric device delivered a high energy density of 14 WhKg^−1^ at a power density of 16.8 kWKg^−1^.

### 4.2 Catalysis

Mo borides and 2D MBenes have shown promising electrocatalytic activities toward various reactions due to the broad specific surface area, diverse surface chemical composition, and metallic conductivity.^[^ ^1^, ^2^, ^25^ ^]^

#### 4.2.1 Hydrogen Evolution Reaction (HER)

In recent years, HER has become one of the most studied research topics due to its highly promising technological characteristics for sustainable and large‐scale hydrogen production capability. A comparative study of the HER activity of Mo_2_B, α‐MoB, β‐MoB, and MoB_2_ demonstrated that MoB_2_ and β‐MoB exhibits excellent activity whereas the molybdenum‐rich Mo_2_B shows significantly lower activity.^[^ ^19^, ^49^ ^]^ But in another report, the CVD‐grown Mo‐rich ultrathin Mo_3_B films of 6.48 nm thickness on Mo foils exhibited fantastic stability and a lower Tafel slope of 52 mVdec^−1^.^[^ ^5^ ^]^ Theoretical studies showed the metallic nature and the faster electron transport along the active edges of the thin films helped to achieve better catalytic properties. The density functional theory (DFT) calculations on free energy revealed that the active sites in the graphene‐like B‐layer contribute to the high HER activity as compared to the phosphorene‐like B‐layer in Mo_2_B_4_.^[^ ^21^ ^]^ Further, comparative theoretical studies on the HER activity confirmed that the performance increases with increasing boron content from Mo_2_B (no B─B bonds are less active) to α‐MoB and β‐MoB (zigzag boron chains are intermediate active) and MoB_2_ (planar graphene‐like boron layers are more active).^[^ ^73^ ^]^ Further, it is demonstrated that the (001) boron layer in hexagonal MoB_2_ (α‐MoB_2_) is the most active (with performance in the range of Pt (111) surface and the puckering this flat boron layer to the chain‐like configuration (phosphorene like layer; β‐MoB_2_ or Mo_2_B_4_) leads to a reduction in the activity. The HER activities of various molybdenum‐based borides produced through various techniques are listed in **Table** **2**.^[^ ^21^, ^22^, ^74^, ^75^, ^76^, ^77^, ^78^, ^79^ ^]^ Recently, 2D MBenes prepared by partial etching of Al from MoAlB have been reported to show promising HER activity.^[^ ^55^, ^74^, ^80^, ^81^, ^82^ ^]^The overpotential of the partially etched MoAl_1‐x_B is reported to decrease by 99 mV (i.e., 301 mV at 10 mAcm^−2^) as compared to the non‐etched crystals due to the increase in the surface area of exposed catalytically active basal planes.^[^ ^82^ ^]^ Recent literature suggests that both α‐MoB_2_ and β‐MoB_2_ show promising HER activity in both acidic and alkaline media with good cycling stability.^[^ ^18^, ^75^, ^83^, ^84^ ^]^ Gong's group showed that α‐MoB_2_ and β‐MoB_2_ nanosheets deliver low overpotential of 124 and 187 mV at 10 mAcm^−2^ with Tafel slope 63 and 49.3 mVdec^−1^, respectively.^[^ ^18^, ^52^ ^]^ Mo_4/3_B_2‐x_T_z_ boridene prepared from borides ((Mo_2/3_Y_1/3_)_2_AlB_2_ and ((Mo_2/3_Sc_1/3_)_2_AlB_2_) by selective etching showed an onset potential of 0.15 V with respect to the reversible hydrogen electrode.^[^ ^85^ ^]^

**Table 2:** Recent literature on the HER applications of molybdenum borides and MBenes.

^[^ ^78^ ^]^ Catalyst	Method of preparation	Overpotential	Tafel slope (mVdec^−1^)	Ref.
Mo_3_B film (6.48 nm)	CVD	249 mV @ 20 mAcm^−2^	52	[6]
Mo_2_B_4_ powder	High temperature (1100° C) sintering	270 mV @ 3.5 mAcm^−2^	80	[21]
Downsized Mo_2_B_5_ powder (≈ 200 nm)	Downsized by electrochemical approach	740 mV @ 10 mAcm^−2^	118.4	[22]
MoB	Commercial powder (High temperature)	301 mV @ 10 mAcm^−2^	55	[74]
α‐MoB_2_	Molten salt method	124 mV @ 10 mAcm^−2^	63	[18]
β‐MoB_2_	Molten salt method	187 mV @ 10 mAcm^−2^	49.3	[52]
MoB_2_ nanoparticles	Redox‐assisted solid‐state meta‐synthesis reaction	154 mV @ 10 mAcm^−2^	49	[75]
Mo_0.9_Ni_0.1_ B_2_	Molten salt technique	222 mV @ 10 mAcm^−2^	68.4	[76]
Mo_2_NiB_2_	High temperature (1100 °C) vacuum annealing	160 mV @ 10 mAcm^−2^	71	[77]
α‐Mo_0.7_W_0.3_B_2_	Arc melting method	201 mV @ 10 mAcm^−2^	63.8	[78]
Pt‐MoAl _1‐x_B	Selective Al etching and chemical method	32 mV @ 10 mAcm^−2^	83.6	[55]
MoB/g‐C_3_N_4_	Physical mixing of commercial MoB and g‐C_3_N_4_ (prepared by thermal polymerization)	152 mV @ 20 mAcm^−2^	46	[79]

**Figure 8:**
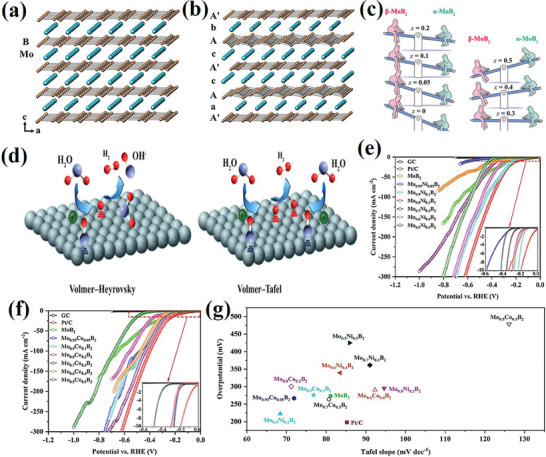
a) Crystal structures of AlB_2_‐type with flat boron layers (α‐MoB_2_), b) Crystal structures of rhombohedral R3m (β‐MoB_2_), c) Schematic represents the modification of the MoB_2_ structure with varying substitution content (x), d) Volmer−Heyrovsky and Volmer−Tafel mechanisms of HER in alkaline solution, e) Linear sweep voltammetry (LSV) curves of Ni‐substituted MoB_2_ 1.0 m KOH, f) LSV curves of Co‐substituted MoB_2_ 1.0 m KOH, g) A comparison of the overpotential (at 10 mAcm^−2^) and Tafel slope on various HER catalysts. Reprinted with permission.^[^ ^76^ ^]^ Copyright 2022. American Chemical Society.

Tailoring the electronic structure by alternating crystal structure and incorporation of heteroatom lead to the reduction of ∆G_H_* and thereby improve the HER activity of molybdenum borides.^[^ ^76^, ^78^, ^86^, ^87^ ^]^ Peighmbardoust et al. demonstrated that by doping transition metals to MoB_2_ with varied concentrations (TM = Ni, Co; x = 0, 0.05, 0.1, 0.2, 0.3, 0.4, and 0.5), HER activity can be tuned.^[^ ^76^ ^]^ Mo_0.9_Ni_0.1_B_2_ afforded 10 mAcm^−2^ at a low overpotential of 220 mV and the assembled Mo_0.9_Ni_0.1_B_2_ (cathode)ǁMo_0.8_Co_0.2_B_2_ (anode) couple demanded 1.75 V to produce 10 mAcm^−2^ which was close to the value of the state‐of‐the‐art Pt/CǁRuO_2_ pair with Faradaic efficiency ≈80% (**Figure** **8**). Park et al. showed that W doping in α‐MoB_2_ promoted hydrogen generation by facilitating bonding between hydrogen atoms in contrast to Mo. In another report, Dutta et al. showed Ni substituted cobalt molybdenum boride nanosheets show superior HER performance with a low overpotential of 69 mV at 10 mAcm^−2^ and Tafel slope of 76.3 mVdec^−1^ in alkaline medium.^[^ ^88^ ^]^ Recently, it was reported that by minimum amount of Pt single atom catalyst loading to partially etched MoAl_1‐x_B, the HER activities impressively enhanced with a low overpotential of 32 mV and 18 mV at 10 mAcm^−2^ in alkaline and acid media.^[^ ^55^ ^]^ Zhang et al. demonstrated that by forming Schottky junctions of metallic MoB with n‐type semiconductor g‐C_3_N_4_, the HER catalytic activities can be optimized.^[^ ^79^ ^]^ The MoB/g‐C_3_N_4_ Schottky catalyst exhibited superior HER activity with a low Tafel slope of 46 mVdec^−1^ and a high exchange current density of 17 µAcm^−2^.

#### 4.2.2 Nitrogen, Carbon Dioxide Reduction Reactions

**Figure 9:**
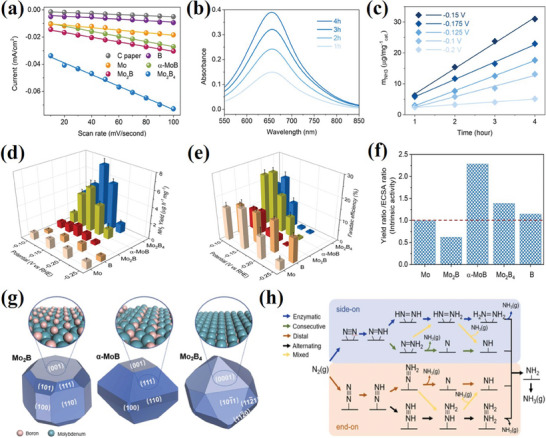
a) The cathodic charging currents of C paper, Mo, B, Mo_2_B, α‐MoB, Mo_2_B_4_ at 0.15 V versus RHE as a function of scan rates; b) Ultraviolet‐visible spectra of the electrolyte stained with indophenol indicator by using Mo_2_B_4_ as a catalyst; c) Ammonia concentration in electrolyte as a function of time using Mo_2_B_4_ as a catalyst in N_2_; d) Ammonia production yields; e) Faradaic efficiencies; f) The intrinsic activity of Mo, Mo_2_B, α‐MoB, Mo_2_B_4_, and B in terms of Yield ratio/ Electrochemically active surface area ratio (with respect to Mo); g) Wulff shapes of Mo_2_B α‐MoB, Mo_2_B_4_ determined by DFT calculations and corresponding most energetically stable surface structures; h) Scheme for the possible reaction mechanism of eNNR. Reprinted with permission.^[^ ^92^ ^]^ Copyright 2023. Elsevier.

Design and synthesis of efficient catalyst for electrochemical nitrogen reduction reaction (eNRR) with less energy consumption, lower CO_2_ emission, high selectivity, and high NH_3_ yield have gained tremendous interest.^[^ ^89^, ^90^, ^91^ ^]^ By considering several advantages and better electrocatalytic properties, Peng et al. investigated molybdenum borides with various Mo‐B stoichiometry ratios (Mo_2_B, α‐MoB, Mo_2_B_4_).^[^ ^92^ ^]^ Mo_2_B_4_‐based catalysis showed the highest NH_3_ yield of 7.65 µgh^−1^/mg at −0.15 V with Faradaic efficiency (FE) of 12.47%, whereas α‐MoB exhibited the fastest eNRR reaction with a higher FE of 17.7% (**Figure** **9**). Further, DFT calculations revealed that due to the intermediate B content in α‐MoB, the reaction intermediates were not able to bind not too weekly or too strongly, which helped to achieve the highest activity of eNRR. Similarly, CrB, MnB, MoB, HfB, and WB were employed as model catalysts to elucidate the associative and dissociative mechanisms of NO_x_ RR. The hydrogenation process involved two pathways: associative, where N─O bond activation occurred after adsorption, and dissociative, where immediate N─O bond breaking led to the formation of isolated N and O atoms on MBenes. This eventually converted into NH_3_ and H_2_O with the addition of H^+^ + e^−^.^[^ ^93^ ^]^

The potential of four 2D MBene nanosheets as catalysts for CO_2_ reduction is quite notable. Mo_2_B_2_ and Cr_2_B_2_ emerged as promising candidates with notable catalytic selectivity, attributed to their subpar performance in HER and a low‐limit potential for CO_2_ reduction.^[^ ^94^, ^95^ ^]^ The study revealed that among the MBenes considered, Mo_2_B_2_ and Cr_2_B_2_ maintained a lower limit potential, recording values of −0.45 and −0.5 eV, respectively. Notably, the analysis of Gibbs energy indicated that CHO formation exhibited the highest increase on all MBenes. Likewise, Xioa et. al. identified C1 hydrocarbon products, including CH_4_, CH_3_OH, HCHO, CO, and HCOOH, suggesting that these MBenes exhibit high stability, catalytic activity, and selectivity in the context of CO_2_ reduction.^[^ ^96^ ^]^ MoB is evaluated as a CO_2_ reduction catalyst, outperforming traditional Mo_2_C due to its metallic nature and excellent electrical conductivity. It effectively activates CO_2_ with higher interaction energy (−3.64 eV), resulting in significant charge transfer. MoB exhibits superior catalytic selectivity by inhibiting the hydrogen evolution reaction and displaying low reaction energy. Particularly, at potentials below −0.62 V, MoB promotes a high‐throughput CO_2_ reduction process, favoring CH_4_ production.^[^ ^97^, ^98^ ^]^

### 4.3 Biochemical Sensing and Surface‐Enhanced Raman Spectroscopy (SERS)

**Figure 10:**
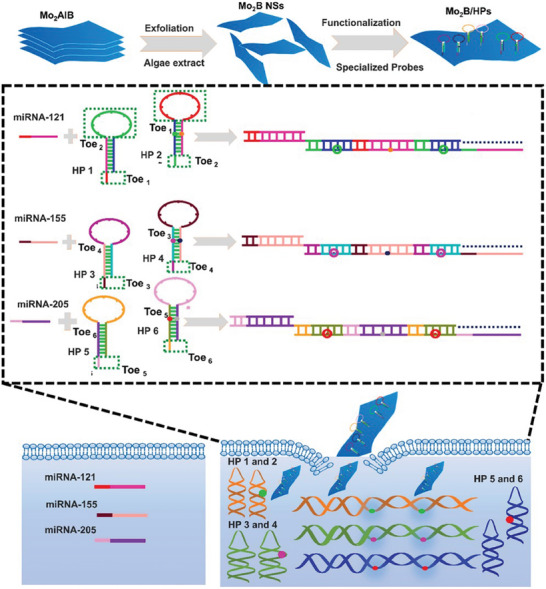
a) Schematic presentation of Mo_2_B nanosheets‐based fluorescence quenching platform for imaging multiple miRNAs in living cells using hybridization chain reaction amplification. Reprinted with permission.^[^ ^99^ ^]^ Copyright 2022. Elsevier.

The combination of the unique optical, electronic, and catalytic properties of molybdenum boride has shown promising performance for its use in biomedical applications for miRNA detection.^[^ ^99^ ^]^ Zada et al. demonstrated the ability for the monitoring of miRNA expression in living cells by Mo_2_B nanosheets (**Figure** **10**). Mo_2_B nanosheets displayed strong interaction with the hairpin probes (HPs), ssDNA loop, and excellent multiple fluorescence quenching performance with ultra‐low background signal. The biomarkers imaging successfully monitored the expression change of miRNAs in cancer cells which holds a promising role in biological and biomedical research.

Pristine MBenes served as favorable adsorbents for capturing NO, SO, and CO. Conversely, functionalized MBenes‐NH_3_ systems exhibited moderate adsorption energies, indicating excellent sensitivity (< 0.5 eV) for NH_3_ gas detection in both phases.^[^ ^100^ ^]^ The optimization of fabrication parameters, functionalization strategies, and sensor configurations is essential for enhancing sensitivity while maintaining robustness and reliability in real‐world applications. Specifically, 2H‐Mo_2_BH_2_ demonstrated higher CT (−0.11e) and appropriate adsorption energy (−0.30 eV), resulting in a shorter recovery time of 12 µs. Additionally, density of states calculations indicated that the electrical conducting properties of MBenes make them well‐suited for efficient NH detection with a brief recovery time.

Due to the multiple crystallographic arrangements, structural transformation, and metallic behavior, MBenes emerge as the ideal candidate for SERS due to the efficient photoinduced charge transfer (PICT) process between MBenes and the molecular level of the probe molecules.^[^ ^101^ ^]^ 2D Mo_4/3_B_2_ MBene displayed higher SERS performance than majority of the semiconductor SERS substrates with a Raman enhancement factor of 3.88 × 10^6^ and ultra‐low detection limit of 1 × 10^−9^ M. DFT calculations confirmed that MBenes have the capability to achieve ultrahigh SERS sensitivity due to the availability of abundant electronic density of states near the Fermi level as compared to the bulk MoB.

### 4.4 Tribology and Lubrication

The application of efficient lubrication materials has an important role in different sectors such as aerospace, automotive, and electrical systems.^[^ ^102^, ^103^, ^104^ ^]^ MAB‐phases, known for their ability to form protective tribo‐films, are gaining prominence as ultra‐high‐temperature materials with oxidation resistance and high damage tolerance. They offer potential in high‐temperature tribology, contributing to extending the lifespan of components and reducing environmental impact through the use of solid lubricants. With properties resembling both metals and ceramics, MAB‐phases can be tailored for specific tribo‐couples, as emphasized by Tao et al. in their use of ternary transition metal carbides, nitrides, and borides for protective surface layers in tribological applications.^[^ ^105^ ^]^ To gain insights into potential applications of Fe_2_AlB_2_ and other MAB‐phases, an investigation focused on its high‐temperature flexural and compressive strength, along with thermal shock resistance in water from room temperature (RT) to 1000 °C. The flexural strength remained within a narrow range of 200–250 MPa across this temperature span, while the compressive strength of Fe_2_AlB_2_ gradually decreased from 1992 MPa at room temperature to 1482 MPa at 600 °C. A further temperature increase led to a rapid decline in compressive strength to 245 MPa at 1000 °C, despite the absence of plastic deformation.^[^ ^106^, ^107^ ^]^ Another study explored the friction and wear behavior of Fe_2_AlB_2_ against GCr^15^ steel, finding that the coefficient of friction (COF) decreased with rising sliding speed and normal force.^[^ ^108^ ^]^ Despite variations in wear rates based on sliding speed and normal force, the wear rates ranged from 0.5 to 2.5 × 10^5^ mm^3^(Nm)^−1^, attributed to the high hardness of Fe_2_AlB_2_. Mn_2_AlB_2_, identified as relatively soft for a transition metal boride, exhibited compressive strengths of 1.24 ± 0.1 GPa, indicating exceptional strength.^[^ ^108^ ^]^ 2D MBenes are demonstrated to offer excellent lubrication properties even under high loads.^[^ ^37^, ^104^ ^]^ MBenes‐based solid lubrication showed adequate colloidal stability with a promising reduction in friction and wear by 50% even under a load of 400 mN (contact pressure of 0.8 GPa).

## 5 Conclusions and Future Perspectives

Molybdenum borides have experienced a steady development both as fundamental sciences and as device applications in the past few years. This review presents the latest developments in molybdenum‐based borides and MBenes. These materials are found in multiple crystallographic arrangements and therefore show unique physical and electronic properties. These materials are possible to synthesize with different strategies such as PVD, CVD, element reaction route, molten salt‐assisted, and selective etching methods. In terms of applications, we mainly review the latest advancements in the fields of energy storage, catalysis, biosensors, and biomedical devices, as well as tribology and lubrication. According to studies, characteristics like large surface area, active edge sites, good conductivity, appreciable stability, and flexibility make them advantageous in achieving superior performance.

**Figure 11:**
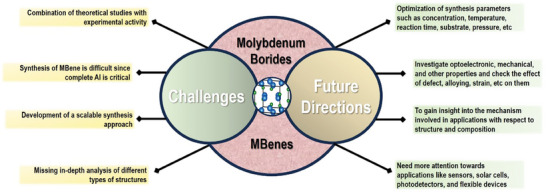
Schematic representation of current challenges and future perspectives for molybdenum‐based borides and MBenes.

Even though significant attempts have been made to better comprehend molybdenum boride, further research is still required (**Figure** **11**). Since total Al etching has not yet been achieved, the synthesis of MBene is challenging. The processes involved in etching techniques need drastic improvement. The understanding of structures and compositions of molybdenum‐based boron compounds will help to prepare the structures, characteristics, and functionalities. The optimization of synthesis parameters such as concentration, temperature, reaction time, substrate, pressure, etc. requires more focus. Tailoring the properties of borides with a controlled synthesis manner can provide an opportunity to fine‐tune the functionality of devices in a variety of prospective applications. The development of a scalable synthesis approach will be critical in establishing commercial uses of molybdenum boride materials. Layer and thickness‐dependent studies may provide chances for device performance improvement. Also, there are several engineering approaches such as defect, strain, alloying, heterostructure fabrication, etc. that can influence the properties of materials. Though molybdenum borides have shown excellent performances in many applications, their performance is still far behind compared to other nanomaterials. The research of applications like a variety of sensors, solar cells, photodetectors, and electrocatalytic reactions is still in the early stages of research and requires more focus. There is a need for more optimization and in‐depth analysis of numerous parameters involved in the application studies. Attention should be given to gain insight into the mechanism of molybdenum borides in different applications. The In situ characterization studies can be useful to gain insights into the structure. This will help to design or engineer the structure and composition of these materials as per the demand of a particular device. The study of these materials' properties is still based on theoretical hypotheses and missing experimental support for that. Thus, the combination of theoretical predictions and experimental outcomes can bring fruitful outcomes in terms of improving application performance. The progress being made in the research of molybdenum boride materials is in the primary stage, however, it appears that these materials have a bright future for variety of applications.

## Conflict of interest

The authors declare no conflict of interest.

## References

[1] J. Chen , Y. Lin , H. Wang , J. Li , S. Liu , J.‐M. Lee , Q. Zhao , Adv. Funct. Mater. 2023, 33, 2210236.

[2] B. Zhang , J. Zhou , Z. Sun , J. Mater. Chem. A 2022, 10, 15865.

[3] H. Chen , X. Zou , Inorg. Chem. Front. 2020, 7, 2248.

[4] Z. Pu , T. Liu , G. Zhang , X. Liu , M. A. Gauthier , Z. Chen , S. Sun , Small Methods 2021, 5, 2100699. 10.1002/smtd.202100699 34927953

[5] X. Wang , G. Tai , Z. Wu , T. Hu , R. Wang , J. Mater. Chem. A 2017, 5, 23471.

[6] S. Carenco , D. Portehault , C. Boissière , N. Mézailles , C. Sanchez , Chem. Rev. 2013, 113, 7981. 23767879 10.1021/cr400020d

[7] Chinese Journal of Structural Chemistry, 2022, 41, 2209008.

[8] B. Albert , H. Hillebrecht , Angew. Chem., Int. Ed. 2009, 48, 8640. 10.1002/anie.200903246 19830749

[9] V. G. Nair , M. Birowska , D. Bury , M. Jakubczak , A. Rosenkranz , A. M. Jastrzębska , Adv. Mater. 2022, 34, 2108840. 10.1002/adma.202108840 35506196

[10] T. Xu , Y. Wang , Z. Xiong , Y. Wang , Y. Zhou , X. Li , Nano‐Micro Lett. 2022, 15, 6. 10.1007/s40820-022-00976-5 PMC9727130 36472760

[11] X. Zhang , A. Chen , L. Chen , Z. Zhou , Adv. Energy Mater. 2022, 12, 2003841.

[12] G. Akopov , M. T. Yeung , R. B. Kaner , Adv. Mater. 2017, 29, 1604506. 10.1002/adma.201604506 28323358

[13] W. Hua , H.‐H. Sun , F. Xu , J.‐G. Wang , Rare Met. 2020, 39, 335.

[14] S. K. Mellerup , S. Wang , Chem. Soc. Rev. 2019, 48, 3537. 31070642 10.1039/c9cs00153k

[15] D. Rhodes , S. H. Chae , R. Ribeiro‐Palau , J. Hone , Nat. Mater. 2019, 18, 541. 31114069 10.1038/s41563-019-0366-8

[16] F. Guo , Y. Wu , H. Chen , Y. Liu , L. Yang , X. Ai , X. Zou , Energy Environ. Sci. 2019, 12, 684.

[17] Y. Wang , H. Zhang , S. Jiao , K.‐C. Chou , G.‐H. Zhang , J. Am. Ceram. Soc. 2020, 103, 2399.

[18] X. Liu , Y. Gong , ACS Appl. Nano Mater. 2022, 5, 10183.

[19] H. Park , A. Encinas , J. P. Scheifers , Y. Zhang , B. P. T. Fokwa , Angew. Chem., Int. Ed. 2017, 56, 5575. 10.1002/anie.201611756 28394098

[20] Y. Chen , G. Yu , W. Chen , Y. Liu , G.‐D. Li , P. Zhu , Q. Tao , Q. Li , J. Liu , X. Shen , H. Li , X. Huang , D. Wang , T. Asefa , X. Zou , J. Am. Chem. Soc. 2017, 139, 12370. 28686430 10.1021/jacs.7b06337

[21] H. Park , Y. Zhang , J. P. Scheifers , P. R. Jothi , A. Encinas , B. P. T. Fokwa , J. Am. Chem. Soc. 2017, 139, 12915. 28871784 10.1021/jacs.7b07247

[22] Y. Wang , C. C. Mayorga‐Martinez , X. Chia , Z. Sofer , N. M. Latiff , M. Pumera , ACS Sustainable Chem. Eng. 2019, 7, 12148.

[23] R. F. Zhang , D. Legut , Z. J. Lin , Y. S. Zhao , H. K. Mao , S. Veprek , Phys. Rev. Lett. 2012, 108, 255502. 23004618 10.1103/PhysRevLett.108.255502

[24] A. T. Lech , C. L. Turner , R. Mohammadi , S. H. Tolbert , R. B. Kaner , Proc. Natl. Acad. Sci. USA 2015, 112, 3223. 25733870 10.1073/pnas.1415018112 PMC4371990

[25] X. Qian , J. Fang , J. Xia , G. He , H. Chen , Int. J. Hydrogen Energy 2023, 48, 26084.

[26] D. V. Rybkovskiy , A. G. Kvashnin , Y. A. Kvashnina , A. R. Oganov , J. Phys. Chem. Lett. 2020, 11, 2393. 32125852 10.1021/acs.jpclett.0c00242

[27] J. Jin , U. Schwingenschlögl , npj 2D Mater. Appl. 2022, 6, 49.

[28] C. Avcıoğlu , S. Avcıoğlu , Materials 2023, 16, 6496.37834632

[29] Y. Cheng , J. Mo , Y. Li , Y. Zhang , Y. Song , Phys. Chem. Chem. Phys. 2021, 23, 6613. 33705501 10.1039/d0cp06405j

[30] Q. Tao , X. Zhao , Y. Chen , J. Li , Q. Li , Y. Ma , J. Li , T. Cui , P. Zhu , X. Wang , RSC Adv. 2013, 3, 18317.

[31] Z. Guo , J. Zhou , Z. Sun , J. Mater. Chem. A 2017, 5, 23530.

[32] Z. Wang , S. W. Fan , H. G. Piao , Z. S. Lu , Appl. Surf. Sci. 2021, 538, 148026.

[33] L. Yan , T. Bo , P.‐F. Liu , B.‐T. Wang , Y.‐G. Xiao , M.‐H. Tang , J. Mater. Chem. C. 2019, 7, 2589.

[34] Z. Meng , Direct synthesis of magnetic bimetallic alloy nanoparticles from organometallic precursors and their applications, 2016.

[35] H. E. Çamurlu , J. Alloys Compd. 2011, 509, 5431.

[36] C. L. Yeh , W. S. Hsu , J. Alloys Compd. 2008, 457, 191.

[37] Q. Tao , Y. Chen , M. Lian , C. Xu , L. Li , X. Feng , X. Wang , T. Cui , W. Zheng , P. Zhu , Chem. Mater. 2019, 31, 200.

[38] P. R. Jothi , K. Yubuta , B. P. T. Fokwa , Adv. Mater. 2018, 30, 1704181. 10.1002/adma.201704181 29457282

[39] D. H. Nguyen , M. C. Ngo , Y. Tokoi , T.‐M.‐D. Do , T. Nakayama , H. Suematsu , K. Niihara , J. Am. Ceram. Soc. 2021, 104, 4351.

[40] F. Qi , Y. Chen , B. Zheng , J. Zhou , X. Wang , P. Li , W. Zhang , Mater. Lett. 2016, 184, 324.

[41] L. Tao , L. Han , Q. Yue , B. Yao , Y. Yang , N. Huo , R. Soc. Open Sci. 2021, 8, 210554. 34430047 10.1098/rsos.210554 PMC8355690

[42] R. Sahu , D. Bogdanovski , J.‐O. Achenbach , S. Zhang , M. Hans , D. Primetzhofer , J. M. Schneider , C. Scheu , Nanoscale 2021, 13, 18077. 34726227 10.1039/d1nr05712j

[43] R. Sahu , D. Bogdanovski , J.‐O. Achenbach , J. M. Schneider , C. Scheu , Nanoscale 2022, 14, 2578. 35107473 10.1039/d1nr07792a

[44] M. Ozkan , K. A. M. Quiros , J. M. Watkins , T. M. Nelson , N. D. Singh , M. Chowdhury , T. Namboodiri , K. R. Talluri , E. Yuan , Chem 2024, 10, 443.

[45] D. Wu , X. Han , C. Wu , Y. Song , J. Li , Y. Wan , X. Wu , X. Tian , J. Phys. Chem. Lett. 2024, 15, 1070. 38261575 10.1021/acs.jpclett.3c02786

[46] W. Xiong , X. Feng , Y. Xiao , T. Huang , X. Li , Z. Huang , S. Ye , Y. Li , X. Ren , X. Wang , X. Ouyang , Q. Zhang , J. Liu , Chem. Eng. J. 2022, 446, 137466.

[47] Z. Cai , B. Liu , X. Zou , H.‐M. Cheng , Chem. Rev. 2018, 118, 6091. 29384374 10.1021/acs.chemrev.7b00536

[48] C. Tsakonas , M. Dimitropoulos , A. C. Manikas , C. Galiotis , Nanoscale 2021, 13, 3346. 33555274 10.1039/d0nr07330j

[49] J. Si , J. Yu , H. Lan , L. Niu , J. Luo , Y. Yu , L. Li , Y. Ding , M. Zeng , L. Fu , J. Am. Chem. Soc. 2023, 145, 3994. 10.1021/jacs.2c11139 36706380

[50] G. Gouget , D. P. Debecker , A. Kim , G. Olivieri , J.‐J. Gallet , F. Bournel , C. Thomas , O. Ersen , S. Moldovan , C. Sanchez , S. Carenco , D. Portehault , Inorg. Chem. 2017, 56, 9225. 28737907 10.1021/acs.inorgchem.7b01279

[51] D. Portehault , S. Devi , P. Beaunier , C. Gervais , C. Giordano , C. Sanchez , M. Antonietti , Angew. Chem., Int. Ed. 2011, 50, 3262. 10.1002/anie.201006810 21432954

[52] X. Liu , Y. Gong , Inorg. Chem. 2021, 60, 18075. 34752079 10.1021/acs.inorgchem.1c02684

[53] K. Kim , C. Chen , D. Nishio‐Hamane , M. Okubo , A. Yamada , Chem. Commun. 2019, 55, 9295. 10.1039/c9cc03855h 31232419

[54] L. T. Alameda , P. Moradifar , Z. P. Metzger , N. Alem , R. E. Schaak , J. Am. Chem. Soc. 2018, 140, 8833. 29906120 10.1021/jacs.8b04705

[55] S. J. Park , T. H. Nguyen , D. T. Tran , V. A. Dinh , J. H. Lee , N. H. Kim , Energy Environ. Sci. 2023, 16, 4093.

[56] J. Zhou , J. Palisaitis , J. Halim , M. Dahlqvist , Q. Tao , I. Persson , L. Hultman , P. O. Å. Persson , J. Rosen , Science 2021, 373, 801. 34385398 10.1126/science.abf6239

[57] A. Majed , M. Torkamanzadeh , C. F. Nwaokorie , K. Eisawi , C. Dun , A. Buck , J. J. Urban , M. M. Montemore , V. Presser , M. Naguib , Small Methods 2023, 7, 2300193. 10.1002/smtd.202300193 37199143

[58] A. Patra , M. Shaikh , S. Ghosh , D. J. Late , C. S. Rout , Sustainable Energy Fuels 2022, 6, 2941.

[59] A. Patra , C. S. Rout , Energy Storage 2023, 5, e411.

[60] A. Sharma , S. Bisoyi , A. Patra , G. K. Pradhan , C. S. Rout , ACS Appl.Nano Mater. 2022, 5, 17526.

[61] A. Patra , P. Mane , K. Pramoda , S. Hegde , B. Chakraborty , C. S. Rout , J. Energy Storage 2023, 68, 107825.

[62] S. Radhakrishnan , A. Patra , G. Manasa , M. A. Belgami , S. M. Jeong , C. S. Rout , Adv. Sci. 2024, 11, 2305325. 10.1002/advs.202305325 PMC10811497 38009510

[63] J. Jia , B. Li , S. Duan , Z. Cui , H. Gao , Nanoscale 2019, 11, 20307. 31633716 10.1039/c9nr05708k

[64] X.‐H. Zha , P. Xu , Q. Huang , S. Du , R.‐Q. Zhang , Nanoscale Adv. 2020, 2, 347. 36133999 10.1039/c9na00610a PMC9417839

[65] G. Barik , S. Pal , Phys. Chem. Chem. Phys. 2023, 25, 17667. 37366646 10.1039/d3cp01189e

[66] T. Bo , P.‐F. Liu , J. Zhang , F. Wang , B.‐T. Wang , Phys. Chem. Chem. Phys. 2019, 21, 5178. 30775754 10.1039/c9cp00012g

[67] Y. Xiao , Y. Li , Z. Guo , C. Tang , B. Sa , N. Miao , J. Zhou , Z. Sun , Appl. Surf. Sci. 2021, 566, 150634.

[68] J. He , A. Bhargav , A. Manthiram , Adv. Mater. 2020, 32, 2004741. 10.1002/adma.202004741 32864813

[69] Z. Guo , Y. Zhao , Y. Miao , D. Wang , D. Zhang , ACS Appl. Energy Mater. 2022, 5, 11844.

[70] Y. Cheng , Z. Li , Y. Liu , J. Qiu , R. Wang , Y. Shi , X. Niu , B. Tan , ACS Mater. Lett. 2023, 5, 2473.

[71] S. Wei , X. Lai , G. M. Kale , ACS Appl. Mater. Interfaces 2023, 15, 33560. 37403562 10.1021/acsami.3c04301 PMC10360035

[72] S. Vinoth , H. T. Das , M. Govindasamy , S.‐F. Wang , N. S. Alkadhi , M. Ouladsmane , J. Alloys Compd. 2021, 877, 160192.

[73] E. Lee , B. P. T. Fokwa , Acc. Chem. Res. 2022, 55, 56. 34904818 10.1021/acs.accounts.1c00543

[74] H. Vrubel , X. Hu , Angew. Chem., Int. Ed. 2012, 51, 12703. 10.1002/anie.201207111 23143996

[75] P. R. Jothi , Y. Zhang , J. P. Scheifers , H. Park , B. P. T. Fokwa , Sustainable Energy Fuels 2017, 1, 1928.

[76] N. S. Peighambardoust , E. Hatipoglu , U. Aydemir , ACS Sustainable Chem. Eng. 2022, 10, 15909.

[77] A. Saad , Y. Gao , K. A. Owusu , W. Liu , Y. Wu , A. Ramiere , H. Guo , P. Tsiakaras , X. Cai , Small 2022, 18, 2104303. 10.1002/smll.202104303 35142066

[78] H. Park , Y. Zhang , E. Lee , P. Shankhari , B. P. T. Fokwa , ChemSusChem 2019, 12, 3726. 31173670 10.1002/cssc.201901301

[79] Z. Zhuang , Y. Li , Z. Li , F. Lv , Z. Lang , K. Zhao , L. Zhou , L. Moskaleva , S. Guo , L. Mai , Angew. Chem. 2018, 130, 505. 10.1002/anie.201708748 29119647

[80] K. J. Baumler , L. T. Alameda , R. R. Katzbaer , S. K. O'Boyle , R. W. Lord , R. E. Schaak , J. Am. Chem. Soc. 2023, 145, 1423. 36602413 10.1021/jacs.2c12496

[81] N. F. Rosli , M. Z. M. Nasir , N. Antonatos , Z. Sofer , A. Dash , J. Gonzalez‐Julian , A. C. Fisher , R. D. Webster , M. Pumera , ACS Appl. Nano Mater. 2019, 2, 6010.

[82] L. T. Alameda , C. F. Holder , J. L. Fenton , R. E. Schaak , Chem. Mater. 2017, 29, 8953.

[83] F. Guo , Y. Wu , X. Ai , H. Chen , G.‐D. Li , W. Chen , X. Zou , Chem. Commun. 2019, 55, 8627. 10.1039/c9cc03638e 31282506

[84] M. D. Scanlon , X. Bian , H. Vrubel , V. Amstutz , K. Schenk , X. Hu , B. Liu , H. H. Girault , Phys. Chem. Chem. Phys. 2013, 15, 2847. 23338557 10.1039/c2cp44522k

[85] P. Helmer , J. Halim , J. Zhou , R. Mohan , B. Wickman , J. Björk , J. Rosen , Adv. Funct. Mater. 2022, 32, 2109060.

[86] X. Gao , Y. Zhou , Y. Tan , B. Yang , Z. Cheng , Z. Shen , J. Jia , Appl. Surf. Sci. 2019, 473, 770.

[87] F. Dohnal , P. Lazar , ChemPhysChem 2023, 24, 202200824. 10.1002/cphc.202200824 36646517

[88] S. Dutta , H. Han , M. Je , H. Choi , J. Kwon , K. Park , A. Indra , K. M. Kim , U. Paik , T. Song , Nano Energy 2020, 67, 104245.

[89] J. G. Chen , R. M. Crooks , L. C. Seefeldt , K. L. Bren , R. M. Bullock , M. Y. Darensbourg , P. L. Holland , B. Hoffman , M. J. Janik , A. K. Jones , M. G. Kanatzidis , P. King , K. M. Lancaster , S. V. Lymar , P. Pfromm , W. F. Schneider , R. R. Schrock , Science 2018, 360, eaar6611. 29798857 10.1126/science.aar6611 PMC6088796

[90] S. L. Foster , S. I. P. Bakovic , R. D. Duda , S. Maheshwari , R. D. Milton , S. D. Minteer , M. J. Janik , J. N. Renner , L. F. Greenlee , Nat. Catal. 2018, 1, 490.

[91] B. H. R. Suryanto , H.‐L. Du , D. Wang , J. Chen , A. N. Simonov , D. R. MacFarlane , Nat. Catal. 2019, 2, 290.

[92] G. Peng , J.‐W. Zhao , J. Wang , E. Hoenig , S. Wu , M. Wang , M. He , L. Zhang , J.‐X. Liu , C. Liu , Appl. Catal., B 2023, 338, 123020.

[93] A. Hermawan , V. N. Alviani , Wibisono , Z. W. Seh , iScience 2023, 26, 107410. 37593457 10.1016/j.isci.2023.107410 PMC10428125

[94] X. Liu , Z. Liu , H. Deng , J. Phys. Chem. C 2021, 125, 19183.

[95] M. Li , Y. Zhang , D. Gao , Y. Li , C. Yu , Y. Fang , Y. Huang , C. Tang , Z. Guo , ChemPhysChem 2024, 25, 202300837. 10.1002/cphc.202300837 38225754

[96] Y. Xiao , C. Shen , N. Hadaeghi , J. Phys. Chem. Lett. 2021, 12, 6370. 34231363 10.1021/acs.jpclett.1c01499

[97] X. Lu , Y. Hu , S. Cao , J. Li , C. Yang , Z. Chen , S. Wei , S. Liu , Z. Wang , Phys. Chem. Chem. Phys. 2023, 25, 18952. 37409409 10.1039/d2cp05449c

[98] X. Bai , Z. Zhao , G. Lu , J. Phys. Chem. Lett. 2023, 14, 5172. 37253226 10.1021/acs.jpclett.3c00903

[99] S. Zada , H. Lu , W. Dai , S. Tang , S. Khan , F. Yang , Y. Qiao , P. Fu , H. Dong , X. Zhang , Biosens. Bioelectron. 2022, 197, 113815. 34814033 10.1016/j.bios.2021.113815

[100] A. Shukla , G. Sharma , S. Krishnamurty , Appl. Surf. Sci. 2023, 615, 156299.

[101] L. Lan , X. Fan , C. Zhao , J. Gao , Z. Qu , W. Song , H. Yao , M. Li , T. Qiu , Nanoscale 2023, 15, 2779. 36661187 10.1039/d2nr06280a

[102] V. S. Saji , R. M. Cook , Corrosion Protection and Control Using Nanomaterials, Elsevier, Amsterdam, The Netherlands 2012.

[103] Y. Liu , S. Yu , W. Wang , Carbon 2022, 198, 119.

[104] A. Rosenkranz , Y. Liu , L. Yang , L. Chen , Appl. Nanosci. 2020, 10, 3353.

[105] Q. Tao , M. Dahlqvist , J. Lu , S. Kota , R. Meshkian , J. Halim , J. Palisaitis , L. Hultman , M. W. Barsoum , P. O. Å. Persson , J. Rosen , Nat. Commun. 2017, 8, 14949. 28440271 10.1038/ncomms14949 PMC5413966

[106] G. Song , D. Sun , X. He , X. Qi , Y. Zheng , J. Gao , H. Yin , Y. Bai , Ceram. Int. 2020, 46, 19912.

[107] Y. Bai , D. Sun , N. Li , F. Kong , X. Qi , X. He , R. Wang , Y. Zheng , International Journal of Refractory Metals and Hard Materials 2019, 80, 151.

[108] X. Tan , P. Chai , C. M. Thompson , M. Shatruk , J. Am. Chem. Soc. 2013, 135, 9553. 23731263 10.1021/ja404107p

